# Postural Balance Ability and the Effect of Visual Restriction on Older Dancers and Non-Dancers

**DOI:** 10.3389/fspor.2021.707567

**Published:** 2021-09-23

**Authors:** Maria-Elissavet Nikolaidou, Vasilios Karfis, Maria Koutsouba, Arno Schroll, Adamantios Arampatzis

**Affiliations:** ^1^Department of Physical Education and Sport Science, School of Physical Education and Sport Science, National and Kapodistrian University of Athens, Athens, Greece; ^2^Department of Training and Movement Sciences, Humboldt-Universität zu Berlin, Berlin, Germany; ^3^Berlin School of Movement Science, Humboldt-Universität zu Berlin, Berlin, Germany

**Keywords:** falls prevention, physical activity, visual channel, proprioception, aging, biomechanics, dance exercise

## Abstract

Dance has been suggested to be an advantageous exercise modality for improving postural balance performance and reducing the risk of falls in the older population. The main purpose of this study was to investigate whether visual restriction impacts older dancers and non-dancers differently during a quiet stance balance performance test. We hypothesized higher balance performance and greater balance deterioration due to visual restriction in dancers compared with non-dancers, indicating the superior contribution of the visual channel in the expected higher balance performances of dancers. Sixty-nine (38 men, 31 women, 74 ± 6 years) healthy older adults participated and were grouped into a Greek traditional dance group (*n* = 31, two to three times/week for 1.5 h/session, minimum of 3 years) and a non-dancer control group (*n* = 38, no systematic exercise history). The participants completed an assessment of one-legged quiet stance trials using both left and right legs and with eyes open while standing barefoot on a force plate (Wii, A/D converter, 1,000 Hz; Biovision) and two-legged trials with both eyes open and closed. The possible differences in the anthropometric and one-legged balance parameters were examined by a univariate ANOVA with group and sex as fixed factors. This ANOVA was performed using the same fixed factors and vision as the repeated measures factor for the two-legged balance parameters. In the one-legged task, the dance group showed significantly lower values in anteroposterior and mediolateral sway amplitudes (*p* = 0.001 and *p* = 0.035) and path length measured in both directions (*p* = 0.001) compared with the non-dancers. In the two-legged stance, we found a significant vision effect on path length (*p* < 0.001) and anteroposterior amplitude (*p* < 0.001), whereas mediolateral amplitude did not differ significantly (*p* = 0.439) between closed and open eyes. The dance group had a significantly lower CoP path length (*p* = 0.006) and anteroposterior (*p* = 0.001) and mediolateral sway amplitudes (*p* = 0.003) both in the eyes-open and eyes-closed trials compared with the control group. The superior balance performance in the two postural tasks found in the dancers is possibly the result of the coordinated, aesthetically oriented intersegmental movements, including alternations between one- and two-legged stance phases, that comes with dance. Visual restriction resulted in a similar deterioration of balance performance in both groups, thus suggesting that the contribution of the visual channel alone cannot explain the superior balance performance of dancers.

## Introduction

Approximately 30–60% of people over 65 years of age experience unintentional falls at least once a year as a result of a loss of balance (Gill et al., 2005; Rubenstein, 2006). Falls are usually characterized by high incidence, high susceptibility to injury, and the severity of consequences, thus having a tremendous impact on older adults, with grave social and financial ramifications (Tinetti, 2003). Fall prevention in healthy elders can be achieved with a satisfactory level of physical conditioning (Cadore et al., 2014; Hamed et al., 2018) that targets age-related impairments in balance, strength, power, and neuromotor coordination (Bierbaum et al., 2013; Bohm et al., 2020). A recent meta-analysis concluded that exercise reduces the rate of falls by 23% and the number of older people who experience one or more falls by 15% (Sherrington et al., 2019). Multicomponent (i.e., a combination of endurance, muscle strength, balance exercises, and/or flexibility or coordination training) exercise programs are being recommended for older adults because evidence suggests they are an effective approach to reducing the risk of falling (Baker et al., 2007; Bouaziz et al., 2016).

In recent years, dance has emerged as an advantageous exercise modality for enhancing the postural stability of healthy elders. Dance involves highly coordinated intersegmental movements with body rotations around multiple planes and axes with continuous alternations between one and two-legged stance phases, thus resulting in a continuous need for postural control (Guzmán-García et al., 2011; Douka et al., 2019a). Dance exercise interventions of short (i.e., 8–12 weeks) (Granacher et al., 2012; Sofianidis et al., 2017) or long durations that employ different dance styles, such as traditional/folk or Latin, have been shown to significantly improve the balance performances of healthy old adults (Kattenstroth et al., 2010; Serra et al., 2016). Furthermore, dancing provides dancers with musical experience, acoustic stimulation, and rhythmic motor coordination; for that reason, it is considered a sensory-enriched form of physical activity that can trigger the integration of sensorimotor performance with perceptual abilities in the elderly population (Kattenstroth et al., 2010; Douka et al., 2019b).

During dancing, there is a high need to achieve an artistic result through memorized forms of aesthetic body configurations either on an individual level or in relation to other partners. Since the visual system provides direct information on the position of the body and its perception of orientation with respect to its surroundings (Horak et al., 1989; Horak, 2006), dancers are expected to rely strongly on visual inputs for postural regulation. It has been reported that contemporary dancers show better balance performance with eyes open compared with matched control groups of non-dancers (Golomer et al., 1999; Hugel et al., 1999; Pérez et al., 2014). Postural control relies on a synergistic relationship between the visual and neuromotor systems (Bonnet and Baudry, 2016), with postural control being better when the visual task involves gazing toward specific targets while attempting to preserve upright standing. It can, therefore, be argued that dancers who practice in acquiring visual feedback from their partners to achieve dance's aesthetic result while maintaining their own stability, would show an enhanced postural control that depends on visual information compared to non-dancers. Given that postural stability depends on the integrated sensory information processing from the visual, vestibular, and proprioceptive systems (Horak et al., 1989; Peterka, 2002), the deprivation of visual information might be more pronounced in dancers, who practice learning to dance through visual information. However, whether the visual channel is an important contributor to the advantageous postural balance performance of dancers has not yet been investigated.

Postural balance performance is typically assessed on the basis of the center of pressure displacement during quiet standing on a force platform. These derived center of pressure (CoP) values represent the geometrical location of the reaction force vector on the platform. Furthermore, CoP amplitude-based parameters are typically used as primary outcome measures and have been found to relate with age-related differences in balance performance (Prieto et al., 1996). These measures of static postural control are also typically employed in detecting sex-related differences, with some studies reporting greater CoP sway movement in older women (Kim et al., 2010; Riva et al., 2013; Chen et al., 2019), while others found older men to have increased postural sway compared with women (Era et al., 1997; Masui et al., 2005; Puszczalowska-Lizis et al., 2018).

The goal of this study was to investigate if visual restriction impacts older dancers and non-dancers differently during a quiet stance balance performance test. We hypothesized higher balance performance and greater balance deterioration due to visual restriction in dancers compared with non-dancers, indicating the superior contribution of the visual channel in the expected higher balance performances in dancers.

## Methods

### Participants

In this cross-sectional study, a municipal senior club and four clubs instructing Greek traditional dance classes were contacted, with a total of 69 (38 men, 31 women, age: 74 ± 6 years) eligible senior adults volunteering to participate. Inclusion criteria required that the participants were healthy adults that were at least 65 years old. Participants were excluded if they reported a history of neuromuscular diseases, musculoskeletal disorders, cardiovascular or severe systemic diseases, severe arthritis, or if they had been taking any medication for the above diseases in the last 6 months.

The participants were grouped into a control group of non-dancers (*n* = 38, 20 men and 18 women, no systematic exercise history) and an experimental group of dancers (*n* = 31, 18 men and 13 women), whose participants had been exercising with Greek traditional dances at a frequency of two to three times per week for 1.5 h per session and for a minimum of 3 years. The study was approved by the Ethics Committee of the School of Physical Education and Sport Science, National and Kapodistrian University of Athens (approval number: 1152/11-12-2019), and all the participants gave their written informed consent in accordance with the Declaration of Helsinki.

### Assessment of Postural Balance Performance

Balance performance was assessed in one-legged and two-legged quiet stance trials. The participants were tested in spacious, quiet rooms with appropriate light and temperature conditions, with a measuring device located in the middle of the room at an approximate distance of 2–3 m between the walls and the participants. During the assessment of the one-legged trials, the participants had their eyes open, stood barefoot with either their left or right leg on a force plate (Wii, A/D converter, 1,000 Hz, 24-bit resolution; Biovision), and maintained a straight body posture with their arms hanging relaxed on their sides. Their gazes were fixed on an imaginary point on the wall 2–3 m in front of them, while their heads were kept parallel to ground level. The order of the starting leg was randomized.

For the two-legged stance trials, the participants were instructed to keep their feet at hip-width apart and stand as motionless as possible with their eyes open, as described above, and eyes closed. In these trials, one researcher was always situated behind the participants for safety reasons. Two trials were performed per visual condition in a randomized order. The duration of every trial in the postural stability measurements was 20 s with 30 s of rest across trials and 1 min of rest between quiet stance conditions. Off-line, the data were filtered using a second bi-directional order digital low-pass Butterworth filter with a 15-Hz cut-off frequency and analyzed with MATLAB custom-made scripts (R2012a, 64 Bit; Mathworks, Natick, MA, United States).

The data were analyzed from the 1st to the 16th second (Δt = 15 s) of each 20-s trial time. Postural balance performance was determined by the following parameters: (a) CoP path length, defined as the sum of Euclidean distances of adjacent measurement points, and (b) CoP sway amplitude, defined as the range (i.e., from minimum to maximum) of the CoP values in the anteroposterior and mediolateral directions. Body height (Bryant et al., 2005) and mass may affect path length and sway amplitudes; therefore, the determined CoP parameters were normalized to body height and mass, with the normalized values (% of body height per kilogram of body mass) being used for statistical analysis. For the two-legged stance, the average value of the two trials was used for the analysis. On the other hand, for the one-legged stance, the average value of the left and right leg trial was also used for analysis.

### Statistical Analyses

All statistical analyses were performed using SPSS Statistics (Version 17.0). The normal distribution of the CoP data was examined by a Kolmogorov–Smirnov test with Lilliefors correction. The statistical testing of normality failed (*p* values: 0.046–0.007 for the two-legged CoP parameters with eyes open and eyes closed and respective *p* = 0.004 to *p* < 0.0001 for the one-legged parameters with eyes open); however, upon visual inspection with quantile-quantile (Q-Q) plots, the CoP data were normal with slight deviations. A two-way ANOVA with group (non-dancers, dancers) and sex (male, female) as fixed factors was performed to test for possible differences in the anthropometric parameters and the one-legged balance performance parameters. An ANOVA for repeated measures was also performed, with vision (open, closed eyes) as the within-subjects factor and group and sex as between-subjects factors on the two-legged balance performance outcome measures. A Bonferroni-corrected pairwise analysis was conducted in the case of a significant interaction between the factors of vision, group, and sex. The level of significance for all the tests was set at a = 0.05. For the graphical representation of the outcomes, we used boxplots depicting the median and the 5th and 95th percentiles as whiskers.

## Results

Age was not significantly different (*p* = 0.192, $\eta_{\text{p}}^{2}$ = 0.026) between the men and the women, but the men were significantly heavier (*p* = 0.032, $\eta_{\text{p}}^{2}$ = 0.069), taller (*p* < 0.001, $\eta_{\text{p}}^{2}=$ 0.479), and had greater body mass indices (*p* = 0.017, $\eta_{\text{p}}^{2}$ = 0.085) than the women across the groups (Table 1). Age was significantly lower in the dancers compared with the non-dancers (*p* < 0.001, $\eta_{\text{p}}^{2}$ = 0.29), while body mass was significantly higher in the dancers (*p* = 0.006, $\eta_{\text{p}}^{2}$ = 0.11) compared with the non-dancers. Body height was also significantly higher in the dancers compared with the non-dancers (*p* = 0.011, $\eta_{\text{p}}^{2}$ = 0.095), while body mass index did not differ between the two groups (*p* = 0.144). There were no significant sex-by-group interaction effects in any of the anthropometric parameters (Table 1).

**Table 1:** Anthropometric data for the non-dancer (control) and dancer (dance) groups (means ± SD).

	**Control (*****N*** **=** **38)**		**Dance (*****N*** **=** **31)**	
	**Males (*N* = 20)**	**Females (*N =* 18)**	**Males (*N* = 18)**	**Females (*N* = 13)**
Age (yr)^*^	79 ± 6	75 ± 4	70 ± 4	71 ± 4
Body mass (kg)^*^^+^	76.2 ± 12.3	72.0 ± 9.4	84.7 ± 8.7	77.8 ± 10.5
Body height (cm)^*^^+^	167 ± 8	158 ± 4	174 ± 7	160 ± 5
Body mass index (kg/m^2^) ^+^	27.1 ± 3.7	28.9 ± 4.5	28.1 ± 2.4	30.6 ± 3.4

* *Statistically significant group effect (p < 0.05)*.

+ *Statistically significant sex effect (p <0.05)*.

**Figure 1:**
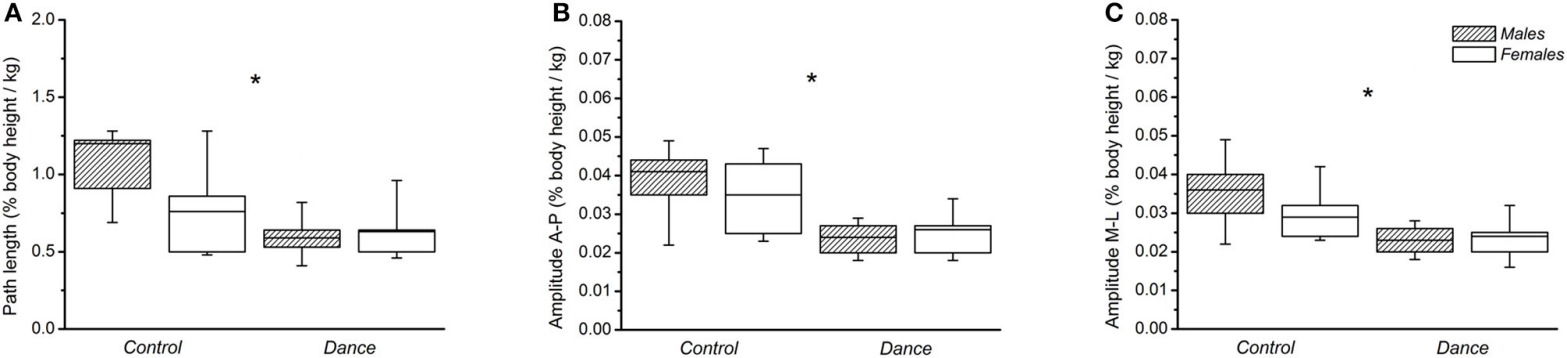
**(A)** Normalized path length, **(B)** center of pressure (CoP)-amplitude in the anteroposterior direction, and **(C)** CoP-amplitude in the mediolateral direction in the one-legged quiet stance trials for men and women in the non-dancer (control) and dancer (dance) groups. *Statistically significant group effect (*p* < 0.05).

In the one-legged quiet stance condition, a significant main effect of group was found for path length (*p* = 0.001, $\eta_{\text{p}}^{2}$ = 0.281) and anteroposterior (*p* = 0.001, $\eta_{\text{p}}^{2}$ = 0.292) and mediolateral (*p* = 0.035, $\eta_{\text{p}}^{2}$ = 0.124) sway amplitudes of the CoP (Figure 1). *Post-hoc* pairwise comparisons showed that the dancers had a significantly smaller path length compared with the non-dancers (*p* = 0.001, Figure 1). The anteroposterior and mediolateral CoP sway amplitudes were also smaller in the dancers compared with the non-dancers (*p* = 0.001 and *p* = 0.035, respectively) (Figure 1). There was no significant main effect of sex on either path length (*p* = 0.079) or anteroposterior (*p* = 0.563) and mediolateral (*p* = 0.208) sway amplitudes across the groups (Figure 1). No significant (*p* > 0.05) sex-by-group interaction effect was found in any of the examined CoP parameter during the one-legged trials.

**Figure 2:**
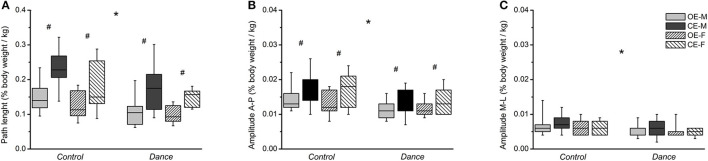
**(A)** Normalized path length, **(B)** CoP-amplitude in the anteroposterior direction, and **(C)** CoP-amplitude in the mediolateral direction in the two-legged quiet stance trials with eyes open (EO) and eyes closed (EC) in the non-dancer (control) and dancer (dance) groups. (M) for men, (F) for women. *Statistically significant group effect (*p* < 0.05), #statistically significant vision effect (*p* < 0.05).

In the two-legged trials, we found a significant effect of group on path length (*p* = 0.006, $\eta_{\text{p}}^{2}$ = 0.111), and anteroposterior (*p* = 0.001, $\eta_{\text{p}}^{2}$ = 0.146) and mediolateral sway amplitudes (*p* = 0.003, $\eta_{\text{p}}^{2}$ = 0.124), with the *post-hoc* pairwise comparisons showing that the dancers had smaller values in these parameters both in the eyes-open and eyes-closed conditions compared with the non-dancers (Figure 2). No significant effect of sex was found for path length (*p* = 0.06) and the anteroposterior (*p* = 0.868) or mediolateral sway amplitudes (*p* = 0.147) of the CoP in the two-legged stance trials. There was a statistically significant main effect of vision on path length (*p* < 0.001, $\eta_{\text{p}}^{2}$ = 0.559) and the anteroposterior sway amplitude of CoP (*p* < 0.001, $\eta_{\text{p}}^{2}$ = 0.339) across the groups (Figure 2). However, there was no significant effect of vision on the mediolateral amplitude of CoP (*p* = 0.439, Figure 2). No significant vision-by-group interaction was also found for either path length (*p* = 0.463) or anteroposterior (*p* = 0.237) and mediolateral sway amplitudes (*p* = 0.74) (Figure 2). Furthermore, there was no statistically significant vision-by-sex interaction for the examined CoP balance parameters (path length, *p* = 0.125; anteroposterior amplitude, *p* = 0.794; mediolateral amplitude, *p* = 0.129) (Figure 2). Likewise, no significant vision-by-group-by-sex interaction was found for either path length (*p* = 0.84) or anteroposterior (*p* = 0.499) and mediolateral sway amplitudes (*p* = 0.814) (Figure 2).

## Discussion

In this study, we found a superior postural balance performance in older dancers during one- and two-legged quiet stance tasks compared with a control group of older non-dancers, supporting the first hypothesis. Furthermore, we found that visual restriction similarly deteriorated balance performance in both groups; therefore, the second hypothesis concerning the greater contribution of the visual channel for postural balance control in older dancers was rejected. Finally, the findings confirmed current reports that found that older men and women have similar balance performances in the investigating balancing tasks when gender differences in anthropometric parameters are accounted for.

During the two-legged quiet stance balance task, we found a decrease in balance performance (i.e., CoP trajectory) in the closed-eyes condition in an average of 52% compared with the open-eyes condition. These findings are in agreement with previous studies that reported an increase in postural sway area and total CoP displacement between 30 and 45% in older adults with vision restrictions (Paulus et al., 1984; Lord et al., 1991; Simoneau et al., 1992). Vision provides direct information on the position and orientation of the body with respect to the environment and, together with proprioception and the vestibular system, is an important mechanism of the neuromotor system in generating accurate motor commands for balance control (Horak et al., 1989). The removal or restriction of these visual inputs deteriorates balance performance during quiet stance (Sarabon et al., 2013; Wiesmeier et al., 2015) and, thus, is a key factor for the overall perception and representation of body positions and movements during postural control. Although there is evidence that the threshold of the visual system for the perception of body sway is lower compared with proprioception (Speers et al., 2002; Doumas et al., 2008) and that older adults use proprioceptive information from the lower limbs more rather than visual and vestibular sensory signals for balance control (Fitzpatrick et al., 1994; Lord and Menz, 2000), visual inputs have been found to detect low-frequency body motions (i.e., 0.01 to 1 Hz) most accurately (Horak et al., 1989), thus contributing to balance performance.

We found a similar increase in CoP trajectory in the closed-eyes condition in both dancers and non-dancers, indicating the negligible effects of exercise history on balance performance deterioration due to visual restrictions. Contemporary dancers exhibited greater balance ability when the visual channel was available (Golomer et al., 1999; Hugel et al., 1999; Pérez et al., 2014) as a result of their increased specialization in tasks where postural control is regulated *via* visual information. Accordingly, we expected a greater contribution of the visual system in older dancers because the execution of artistically oriented intersegmental movements, in combination with the needed movement synchronization and adjustment with other dance partners, challenges the neuromotor system and increases the need for vision in postural control during dancing. Therefore, we expected a greater deterioration of balance performance during the closed-eyes condition in this group. The findings (i.e., similar balance deterioration in both groups with closed-eyes), however, did not confirm a higher contribution of vision in the balance control of dancers; thus, the visual channel cannot explain the superior balance performance of the investigated older dancers. Proprioception due to the lower threshold for the perception of body sway compared with visual and vestibular systems and the key role in postural control (Speers et al., 2002; Doumas et al., 2008) might be a candidate to explain the higher balance performance of older dancers. The probable higher effectiveness of dancers in multisensory integration to control balance and orientation could be further examined in conditions of increasing balance difficulty and differentiating visual cues, as an earlier study showed that the effectiveness of these mechanisms depends on the ability of a participant to identify and exploit appropriate non-visual frames of reference (Isableu et al., 2010).

In recent years, growing research evidence has stressed the importance of dance as an advantageous exercise modality for improving stability performance (Sofianidis et al., 2009, 2017; Granacher et al., 2012; Rehfeld et al., 2017), which consequently reduces the risk of falls in older adults. The one-legged quiet stance is a challenging balance task because the small base of support requires the successful integration of sensory information from the visual, vestibular, and somatosensory systems by the central nervous system to produce appropriate postural responses (Nashner, 1981; Horak, 2006). Greek traditional dancing is characterized by coordinated, rhythmic, aesthetically oriented intersegmental movements with body rotations and the combinations of various steps under musical guidance, resulting in the continuous postural control of the body. In particular, through dancing, the lower extremities are engaged in a continuous alternation of the one- and two-legged stance phases, such as hopping, sideways steps, or crossing one foot over the other, thus favoring the strong sensorimotor control of body sway (Guzmán-García et al., 2011). As a result, dancers become very familiar with weight shifts that constantly challenge their postural control system to maintain equilibrium, as their centers of mass are voluntarily shifted to the limits of stability (Rehfeld et al., 2017). For instance, healthy older adults who have been participating in training interventions (8–12 weeks duration) under various styles of dance, either traditional/folk or modern, were able to achieve relevant improvements in one-legged stance balance performance compared with non-trained peers (Granacher et al., 2012; Rehfeld et al., 2017; Sofianidis et al., 2017). Thus, we can argue that the plethora of high-coordination movement repertoires in dancing, which challenges the human sensorimotor system, can trigger neuromuscular adaptational responses and consequently explain the observed superior balance performance of older dancers.

The findings also revealed no significant sex-related effect on one- and two-legged postural balance performance. Several studies have found that older men exhibit impaired balance performance (i.e., higher CoP sway movement) more than women (Era et al., 1997; Masui et al., 2005; Puszczalowska-Lizis et al., 2018), whereas other studies reported that older women have increased postural sway compared with men (Kim et al., 2010; Riva et al., 2013; Chen et al., 2019). It has also been reported that the smaller body height of women is an important explanatory factor for increased balance performance (Era et al., 1996; Bryant et al., 2005), with the difference between the sexes being largely removed when CoP trajectory was normalized to body height (Bryant et al., 2005). In our investigation, we used normalized body height and mass values to account for the observed greater mass and height in men; therefore, the findings showed that the postural balance performance of older men and women was similar when those parameters were accounted for.

This study has some limitations that should be addressed. The cross-sectional design of the study cannot suppress any selection effects on the balance performance on the investigated dancers (i.e., dancers inherently had greater balance ability and because of that self-selected to become dancers). The main purpose, however, was to examine the deterioration in balance performance due to visual restriction; therefore, the selection bias was reduced. To achieve this, the CoP outcomes were considered for 15 s in each condition. The protocol involved two visual conditions (eyes open and eyes closed) and two stance conditions (two-legged and one-legged); thus, with a longer duration per trial, fatigue would accumulate, most probably for the non-dancers, and could introduce bias into the comparisons between the two groups. Furthermore, the two groups were not exactly matched, as the dancers were heavier, taller, and younger compared with the non-dancers in the control group. The non-dancers were also not included in any systematic exercise programs, indicating generally lower physical activity levels compared with the dancers. Thus, we normalized the assessment parameters to body height and body mass excluding the bias due to body height and body mass; however, the bias of the different ages and physical activity levels remained in this study.

In conclusion, we found superior balance performance in the group of older dancers compared with the control non-dancer group without any systematic exercise experience. The restriction of visual information decreased balance performance similarly in both investigated groups, indicating that the visual channel was not responsible for the superior balance performance found in the dancers. The findings, however, indicate that dancing can be recommended as an advantageous exercise modality in order to improve postural stability as it has the potential to reduce the risk of falls in older adults. We argue that the advanced postural performance of dancers may be triggered by the aesthetically and highly coordinative movement repertoires in dancing that challenge sensorimotor integration.

## Data Availability Statement

The raw data supporting the conclusions of this article will be made available by the authors, without undue reservation.

## Ethics Statement

The studies involving human participants were reviewed and approved by the Ethics Committee of the School of Physical Education and Sport Science of the National and Kapodistrian University of Athens. The patients/participants provided their written informed consent to participate in this study.

## Author Contributions

M-EN and AA conceived the experiments, interpreted the data, and drafted the manuscript. M-EN performed the experiments and analyzed the data. AS and AA substantially contributed to the data analyses. VK and MK made important intellectual contributions during revision. All authors approved the final version of the manuscript and agreed to be accountable for the content of the article.

## Funding

We acknowledge the support of the German Research Foundation (DFG) and the Open Access Publication Fund of Humboldt-Universität zu Berlin.

## Conflict of Interest

The authors declare that the research was conducted in the absence of any commercial or financial relationships that could be construed as a potential conflict of interest.

## Publisher's Note

All claims expressed in this article are solely those of the authors and do not necessarily represent those of their affiliated organizations, or those of the publisher, the editors and the reviewers. Any product that may be evaluated in this article, or claim that may be made by its manufacturer, is not guaranteed or endorsed by the publisher.

## References

[B1] Baker M. K. Atlantis E. FiataroneSingh M. A. (2007). Multi modal exercise programs for older adults. Age Ageing 36, 375–381. 10.1093/ageing/afm054 17537741

[B2] Bierbaum S. Peper A. Arampatzis A. (2013). Exercise of mechanisms of dynamic stability improves the stability state after an unexpected gait perturbation in elderly. Age 35, 1905–1915. 10.1007/s11357-012-9481-z 23054828 PMC3776125

[B3] Bohm S. Mandla-Liebsch M. Mersmann F. Arampatzis A. (2020). Exercise of dynamic stability in the presence of perturbations elicit fast improvements of simulated fall recovery and strength in older adults: a randomized controlled trial. Front. Sports Act Living 27:52. 10.3389/fspor.2020.00052 33345043 PMC7739602

[B4] Bonnet C. T. Baudry S. (2016). Active vision task and postural control in healthy, young adults: Synergy and probably not duality. Gait Posture 48, 57–63. 10.1016/j.gaitpost.2016.04.016 27477709

[B5] Bouaziz W. Lang P. O. Schmitt E. Kaltenbach G. Geny B. Vogel T. (2016). Health benefits of multicomponent training programmes in seniors: a systematic review. Int. J. Clin. Pract. 70, 520–536. 10.1111/ijcp.12822 27291143

[B6] Bryant E. C. Trew M. E. Bruce A. M. Kuisma R. M. E. Smith A. W. (2005). Gender differences in balance performance at the time of retirement. Clin. Biomech. 20, 330–335. 10.1016/j.clinbiomech.2004.11.006 15698707

[B7] Cadore E. L. Pinto R. S. Bottaro M. Izquierdo M. (2014). Strength and endurance training prescription in healthy and frail elderly. Aging Dis. 5, 183–195. 10.14336/AD.2014.0500183 24900941 PMC4037310

[B8] Chen Z. Han J. Waddington G. Adams R. Witchalls J. (2019). Somatosensory perception sensitivity in voluntary postural sway movements: Age, gender and sway effect magnitudes. Exp. Gerontol. 122, 53–59. 10.1016/j.exger.2019.04.013 31029824

[B9] Douka S. Zilidou V. I. Lilou O. Manou V. (2019a). Traditional dance improves the physical fitness and well-being of the elderly. Front. Aging Neurosci. 11:75. 10.3389/fnagi.2019.00075 31024290 PMC6463898

[B10] Douka S. Zilidou V. I. Lilou O. Tsolaki M. (2019b). Greek traditional dances: a way to support intellectual, psychological, and motor functions in senior citizens at risk of neurodegeneration. Front. Aging Neurosci. 11:6. 10.3389/fnagi.2019.00006 30740051 PMC6356054

[B11] Doumas M. Smolders C. Krampe R. T. (2008). Task prioritization in aging: effects of sensory information on concurrent posture and memory performance. Exp. Brain Res. 187, 275–281. 10.1007/s00221-008-1302-3 18273609

[B12] Era P. Avlund K. Jokela J. Gause-Nilsson I. Heikkinen E. Steen B.. (1997). Postural balance and self-reported functional ability in 75-year-old men and women: a cross-national comparative study. J. Am. Geriatr. Soc. 45, 21–29. 10.1111/j.1532-5415.1997.tb00973.x 8994483

[B13] Era P. Schroll M. Ytting H. Gause-Nilsson I. Heikkinen E. Steen B. (1996). Postural balance and its sensory-motor correlates in 75-year-old men and women: a cross-national comparative study. J. Gerontol. A Biol. Sci. Med. Sci. 51, M53–M63. 10.1093/gerona/51a.2.m53 8612104

[B14] Fitzpatrick R. Rogers D. K. McCloskey D. I. (1994). Stable human standing with lower-limb muscle afferents providing the only sensory input. J. Physiol. 480, 395–403. 10.1113/jphysiol.1994.sp020369 7869254 PMC1155855

[B15] Gill T. Taylor A. W. Pengelly A. (2005). A population-based survey of factors relating to the prevalence of falls in older people. Gerontology 51, 340–345. 10.1159/000086372 16110237

[B16] Golomer E. Crémieux J. Dupui P. Isableu B. Ohlmann T. (1999). Visual contribution to self-induced body sway frequencies and visual perception of male professional dancers. Neurosci. Lett. 267, 189–192. 10.1016/s0304-3940(99)00356-0 10381008

[B17] Granacher U. Muehlbauer T. Bridenbaugh S. A. Wolf M. Roth R. Gschwind Y.. (2012). Effects of a salsa dance training on balance and strength performance in older adults. Gerontology 58, 305–312. 10.1159/000334814 22236951

[B18] Guzmán-García A. Johannsen L. Wing M. A. (2011). Dance exercise for older adults: a pilot study investigating standing balance following a single lesson of danzón. Am. J. Dance Ther. 33, 148–156. 10.1007/s10465-011-9114-6

[B19] Hamed A. Bohm S. Mersmann F. Arampatzis A. (2018). Follow-up efficacy of physical exercise interventions on fall incidence and fall risk in healthy older adults: a systematic review and meta-analysis. Sports Med. Open 4:56. 10.1186/s40798-018-0170-z 30547249 PMC6292834

[B20] Horak F. B. (2006). Postural orientation and equilibrium: what do we need to know about neural control of balance to prevent falls? Age Ageing 35, ii7–ii11. 10.1093/ageing/afl077 16926210

[B21] Horak F. B. Shupert C. L. Mirka A. (1989). Components of postural dyscontrol in the elderly: A review. Neurobiol. Aging 10, 727–738. 10.1016/0197-4580(89)90010-9 2697808

[B22] Hugel F. Cadopi M. Kohler F. Perrin P. (1999). Postural control of ballet dancers: a specific use of visual input for artistic purposes. Int. J. Sports Med. 2, 86–92. 10.1055/s-2007-971098 10190767

[B23] Isableu B. Ohlmann T. Cremieux J. Vuillerme N. Amblard B. Gresty M. A. (2010). Individual differences in the ability to identify, select and use appropriate frames of reference for perceptuo-motor control. Neuroscience 169, 1199–1215. 10.1016/j.neuroscience.2010.05.072 20570716

[B24] Kattenstroth J. C. Kolankowska I. Kalisch T. Dinse H. R. (2010). Superior sensory, motor, and cognitive performance in elderly individuals with multi-year dancing activities. Front. Aging Neurosci. 2:31. 10.3389/fnagi.2010.00031 20725636 PMC2917240

[B25] Kim J. W. Eom G. M. Kim C. S. Kim D. H. Lee J. H. Park B. K.. (2010). Sex differences in the postural sway characteristics of young and elderly subjects during quiet natural standing. Geriatr. Gerontol. Int. 2, 191–198. 10.1111/j.1447-0594.2009.00582.x 20100287

[B26] Lord S. R. Clark R. D. Webster I. W. (1991). Postural stability and associated physiological factors in a population of aged persons. J. Gerontol. 46, M69–M76. 10.1093/geronj/46.3.m69 2030269

[B27] Lord S. R. Menz H. B. (2000). Visual contributions to postural stability in older adults. Gerontology 46, 306–310. 10.1159/000022182 11044784

[B28] Masui T. Hasegawa Y. Matsuyama Y. Sakano S. Kawasaki M. Suzuki S. (2005). Gender differences in platform measures of balance in rural community-dwelling elders. Arch. Gerontol. Geriatr. 41, 201–209. 10.1016/j.archger.2005.02.003 16085072

[B29] Nashner L. M. (1981). “Analysis of stance posture in humans,” in Motor Coordination, eds A. L. Towe and E. S. Luschei (Boston, MA: Springer).

[B30] Paulus W. M. Straube A. Brandt T. (1984). Visual stabilization of posture. Physiological stimulus characteristics and clinical aspects. Brain 107, 1143–1163. 10.1093/brain/107.4.1143 6509312

[B31] Pérez R. M. Solana R. S. Barbado Murillo D. Hernández F. J. M. (2014). Visual availability, balance performance and movement complexity in dancers. Gait Posture 4, 556–560. 10.1016/j.gaitpost.2014.06.021 25086798

[B32] Peterka R. J. (2002). Sensorimotor integration in human postural control. J. Neurophysiol. 88, 1097–1018. 10.1152/jn.00605.2001 12205132

[B33] Prieto T. E. Myklebust J. B. Hoffmann R. G. Lovett E. G. Myklebust B. M. (1996). Measures of postural steadiness: differences between healthy young and elderly adults. IEEE Trans. Biomed. Eng. 43, 956–966. 10.1109/10.532130 9214811

[B34] Puszczalowska-Lizis E. Bujas P. Jandzis S. Omorczyk J. Zak M. (2018). Inter-gender differences of balance indicators in persons 60-90 years of age. Clin. Interv. Aging 13, 903–912. 10.2147/CIA.S157182 29785097 PMC5955023

[B35] Rehfeld K. Müller P. Aye N. Schmicker M. Dordevic M. Kaufmann J.. (2017). Dancing or fitness sport? The effects of two training programs on hippocampal plasticity and balance abilities in healthy seniors. Front. Hum. Neurosci. 11:305. 10.3389/fnhum.2017.00305 28674488 PMC5475381

[B36] Riva D. Mamo C. Fanì M. Saccavino P. Rocca F. Momenté M.. (2013). Single stance stability and proprioceptive control in older adults living at home: gender and age differences. Aging Res 2013:561695. 10.1155/2013/561695 23984068 PMC3745841

[B37] Rubenstein L. Z. (2006). Falls in older people: epidemiology, risk factors and strategies for prevention. Age Ageing 35, ii37–ii41. 10.1093/ageing/afl084 16926202

[B38] Sarabon N. Panjan A. Latash M. (2013). The effects of aging on the rambling and trembling components of postural sway: Effects of motor and sensory challenges. Gait Posture 38, 637–642. 10.1016/j.gaitpost.2013.02.007 23454042

[B39] Serra M. M. Castilho Alonso A. Peterson M. Mochizuki L. D'AndréaGreve J. M. Garcez-Leme L. E. (2016). Balance and muscle strength in elderly women who dance Samba. PLoS ONE 11:e0166105. 10.1371/journal.pone.0166105 27906984 PMC5132314

[B40] Sherrington C. Fairhall N. J. Wallbank G. K. Tiedemann A. Michaleff Z. A. Howard K.. (2019). Exercise for preventing falls in older people living in the community. Cochrane Database Syst. Rev. 31:CD012424. 10.1002/14651858.CD012424.pub2 30703272 PMC6360922

[B41] Simoneau G. G. Leibowitz H. W. Ulbrecht J. S. Tyrrell R. A. Cavanagh P. R. (1992). The effects of visual factors and head orientation on postural steadiness in women 55 to 70 years of age. J. Gerontol. 47, M151–M158. 10.1093/geronj/47.5.m151 1512430

[B42] Sofianidis G. Dimitriou A. M. Hatzitaki V. (2017). A Comparative study of the effects of pilates and latin dance on static and dynamic balance in older adults. J. Aging Phys. Act. 25, 412–419. 10.1123/japa.2016-0164 27992251

[B43] Sofianidis G. Hatzitaki V. Douka S. Grouios G. (2009). Effect of a 10-week traditional dance program on static and dynamic balance control in elderly adults. J. Aging Phys. Act. 17:167–180. 10.1123/japa.17.2.167 19451666

[B44] Speers R. A. Kuo A. D. Horak F. B. (2002). Contributions of altered sensation and feedback responses to changes in coordination of postural control due to aging. Gait Posture 16, 20–30. 10.1016/S0966-6362(02)00003-6 12127183

[B45] Tinetti M. E. (2003). Clinical practice: PREVENTING falls in elderly persons. N. Engl. J. Med. 348, 42–49. 10.1056/NEJMcp020719 12510042

[B46] Wiesmeier I. K. Dalin D. Maurer C. (2015). Elderly use proprioception rather than visual and vestibular cues for postural motor control. Front. Aging Neurosci. 7:97. 10.3389/fnagi.2015.00097 26157386 PMC4477145

